# Safety and tolerability of theta burst stimulation vs. single and paired pulse transcranial magnetic stimulation: a comparative study of 165 pediatric subjects

**DOI:** 10.3389/fnhum.2015.00029

**Published:** 2015-02-04

**Authors:** Yaejee H. Hong, Steve W. Wu, Ernest V. Pedapati, Paul S. Horn, David A. Huddleston, Cameron S. Laue, Donald L. Gilbert

**Affiliations:** ^1^College of Medicine, University of CincinnatiCincinnati, OH, USA; ^2^Division of Neurology, Cincinnati Children’s Hospital Medical CenterCincinnati, OH, USA; ^3^Division of Psychiatry, Cincinnati Children’s Hospital Medical CenterCincinnati, OH, USA

**Keywords:** children, youth, transcranial magnetic stimulation, repetitive transcranial magnetic stimulation, theta burst stimulation, safety

## Abstract

**Background**: Although single- and paired-pulse (sp/pp) transcranial magnetic stimulation (TMS) studies are considered minimal risk in adults and children, the safety profile for theta-burst TMS (TBS) is unknown.

**Objective**: In this comparative analysis, we explored the rate, severity, and specific symptoms of TMS-related adverse effects (AEs) between sp/ppTMS and TBS in subjects between ages 6 and 18 years.

**Method**: Data from 165 participants from 2009 to 2014 were analyzed. Assessment of AEs was performed based on baseline and post-TMS administration of a symptom-based questionnaire that rated AEs on a 5-level ordinal scale (minimal, mild, moderate, marked, severe). AE rates and severity were compared using Chi Square or Fisher’s Exact Test depending on data characteristics.

**Result**: Overall, no seizures or severe-rated AEs were reported by 165 pediatric participants. The rate of AE in all TBS sessions was 10.5% (*n* = 76, 95% CI: 4.7–19.7%), whereas the rate of AE in all sp/ppTMS sessions was 12.4% (*n* = 89, 95% CI: 6.3–21.0%). There was no statistical difference in AE rates between TBS and sp/ppTMS (*p* = 0.71). In all sp/ppTMS and TBS sessions, 20 subjects reported a total of 35 AEs, among these 31 (~88.6%) were rated as “minimal” or “mild”. There was no difference in the severity of AE between TBS and sp/ppTMS (*p* = 1.0). Only one of 76 TBS participants reported an AE rated as more than minimal/mild.

**Conclusion**: Our comparative analysis showed that TBS appears to be as safe as sp/ppTMS in terms of AE rate and severity. This report supports further investigation of TBS in children.

## Introduction

Transcranial magnetic stimulation (TMS) is a non-invasive form of brain stimulation that has been increasingly used to develop physiological biomarkers and in therapeutic applications of neurological and psychiatric conditions across a wide range of subjects. Currently, consensus guidelines have suggested that single- and paired-pulse TMS (sp/ppTMS) may be considered as minimal risk in children (Gilbert et al., 2004; Rossi et al., 2009). In contrast, repetitive TMS (rTMS) which may include rapid trains of TMS pulses do not have clear guidelines for use in the pediatric population and carry a potential risk of epileptogenesis (Oberman and Pascual-Leone, 2009). Theta Burst Stimulation (TBS), a type of rTMS, can induce effects on cortical excitability that outlast the stimulation period (Huang et al., 2005). Although TBS and conventional rTMS have been shown to elicit comparable cortical neurophysiologic changes (Zafar et al., 2008; Di Lazzaro et al., 2011), the TBS procedure has two advantages: (1) shorter stimulation duration; and (2) lower stimulation intensity. These features decrease the likelihood of discomfort from TMS pulses, thus making TBS a potentially ideal rTMS protocol to use in pediatric studies.

Several systematic reviews have suggested that TBS is relatively well-tolerated in the adult population including two recent studies that have estimated an approximately 5% rate of adverse events of adults undergoing TBS, which are primarily mild (Oberman et al., 2011; Maizey et al., 2013). To our knowledge, only one serious adverse event (AE), seizure, was reported in a healthy adult male during continuous TBS performed at 100% of resting motor threshold (RMT; Oberman and Pascual-Leone, 2009). In children, we recently reported a total AE rate of 11.6% in 40 children undergoing TBS (Wu et al., 2012).

Future applications of TBS in children as a biomarker or as a therapeutic modality are contingent on a clearer estimate of potential risks of adverse events including sharing sensitive safety data between laboratories (Rossi et al., 2009). In the present report, we compare AE rates between TBS and sp/ppTMS in a cohort of youth over a five year period in a TMS lab within a large stand-alone children’s hospital. We additionally explored the incidence of adverse events across protocol parameters and examined predictors of adverse events.

## Materials and methods

### Participants

Data from 165 unique participants (69 females, 96 males) between ages 6–18 years were analyzed from Institutional Review Board (IRB)-approved protocols which were active in our TMS Lab between 2009 and 2014. Subjects with epilepsy, hearing problems, serious medical condition(s), or implanted medical device(s) were excluded from participation. Recruitment occurred through sub-specialty clinics, hospital wide emails, and from the community. Safety data for TBS was drawn from two studies: (1) TBS technique optimization and biomarker studies which involved healthy and Tourette Syndrome (TS) youths; and (2) a sham-controlled continuous TBS study in TS (Wu et al., 2014). Sp/ppTMS safety data was summarized from studies involving youth with attention deficit hyperactivity disorder (ADHD) and typically developed controls. For the sp/ppTMS studies, ADHD subjects on non-stimulant medications (e.g., atomoxetine) were excluded and those on stimulants were instructed to hold the medication for at least 24 h prior to participation. None of the typically developed controls were on any neuropsychiatric medications at the time of participation. All parent(s)/guardian(s) gave written informed consent for the studies.

### Transcranial magnetic stimulation

Sp/ppTMS was performed with a Magstim 200/Bistim stimulator and a 70 mm figure-8 coil (Magstim Co., Wales, UK). Surface electromyography (EMG) leads were placed over the dominant first dorsal interosseous (FDI) muscle. The coil was placed over the dominant primary motor cortex at the optimal site for obtaining a motor-evoked potential (MEP). RMT and active motor thresholds, cortical silent period, and single and paired pulse amplitudes and ratios were quantified using standard methods, requiring approximately 200 TMS pulses (Rossini et al., 1994; Mills and Nithi, 1997). In paired pulse TMS studies, the intensities of the conditioning and test pulses were predetermined and set at 60% and 120% of RMT respectively. TBS was performed with Magstim SuperRapid2 (Magstim Co., Wales, UK). TBS stimulation intensities ranged from 60–90% RMT. Three pulses were administered at 30 to 50 Hz pulse frequency, 5 Hz burst frequency, with a total number of pulses of either 300 or 600 (Huang et al., 2005). TBS was preceded and followed by spTMS used for post-TBS MEP measurement. This required approximately 200 spTMS pulses. Nine participants received both intermittent and continuous TBS (iTBS, cTBS). TBS administration for the sham controlled trial (Wu et al., 2014) did not involve post TBS assessment of MEP amplitudes.

### Assessment of adverse events

A sixteen-question review of systems (ROS) questionnaire was administered to rate the subjective symptom (headache, scalp pain, arm/hand pain, other pain(s), numbness/tingling, other sensation(s), weakness, loss of dexterity, vision/hearing change(s), ear ringing, nausea/vomiting, appetite loss, rash, skin change(s) or any other symptom(s)) on a scale of 0 to 5 (none, minimal, mild, moderate, marked, severe) prior to any TMS application. At the end of the study after the entire TMS session, this ROS was repeated to detect any AE. The presence of an AE was defined as a positive increase in any of the ROS criteria compared to pre-TMS. The rate of AE was defined as the ratio of sessions with adverse events divided by total sessions. In the Tourette Syndrome study (Wu et al., 2014), patients received two consecutive days of real or sham TBS. Only day 1 AEs were used for data analysis.

### Statistical analysis

*T*-test or Wilcoxon Mann Whitney test was used to compare demographics depending on data distribution. Comparison of AE rates were analyzed using either Chi Square or Fisher’s Exact Test depending on whether any cell of the 2 × 2 table has a count <5. All types of TBS were combined into one group. TS, ADHD, and other motor disorder were considered “affected”. Logistic regression analyses were used to estimate effects of additional predictors. Analyses including power calculations were performed using SAS v9.3 (Cary, NC).

## Results

### Demographics

Demographics and clinical data are shown in Table 1. Among 76 children receiving TBS, 68% were typically developing healthy controls, 25% had a diagnosis of TS, or 7% had other motor disorders. Among 89 participants receiving sp/ppTMS, 21% participants were healthy controls, and 79% participants were affected with ADHD. Of the 24 TBS participants with either TS or other motor disorders, 15 of them were taking neuropsychiatric medications at the time of the TMS session. Collectively, these medications included amitriptyline, atomoxetine, baclofen, citalopram, clonidine, dexmethylphenidate, escitalopram, guanfacine, melatonin, methylphenidate, pimozide, quetiapine, risperidone and sertraline.

**Table 1:** **All participant characteristics and adverse events**.

Participant characteristics	TBS protocols (*n* = 76)	Sp/ppTMS protocols (*n* = 89)	*p*-value
Age, mean (SD)	12.3 (2.9)	10.3 (2.5)	<0.0001
Sex (% male)	57.9%	58.4%	0.63
Diagnosis (% control)	68.4%	21.3%	<0.0001
% Sessions with adverse events (95% CI)	10.5% (4.7–19.7%)	12.4% (6.3–21.0%)	0.71

*CI = confidence interval, SD = standard deviation, TBS = Theta Burst Stimulation, Sp/ppTMS = single and paired pulse transcranial magnetic stimulation*.

### Adverse event rates

AE rates are shown in Table 2. All participants completed sp/ppTMS or TBS sessions without seizures and there were no serious adverse events. There was no statistical difference in AE rates between sp/ppTMS and TBS sessions (*p* = 0.71). There was no difference in frequency of AE between sham and real TBS in the clinical trial. The AE rates were not statistically significant (*p* = 1.0) between cTBS-only (12.5%, *n* = 8) and iTBS-only sessions (11.9%; *n* = 59).

**Table 2:** **Percentages of participants experiencing specific adverse events**.

Symptom	TBS (*n* = 76)	Sp/ppTMS (*n* = 89)
Headache	6.6%	6.7%
Scalp pain	–	4.5%
Arm/hand/other pain	2.6%^*^	2.2%
Numbness/tingling	2.6%	5.6%
Other sensations	2.6%	1.1%^**^
Weakness	1.3%	–
Ringing in ears	–	1.1%^*^
Nausea/vomiting	–	1.1%
Other	1.3%	1.1%

All adverse events were rated minimal to mild except: *one subject each rated “moderate”; **one subject rated “marked”. “Other sensations**” referred to chest pain that was present at baseline due to one subject having an upper respiratory infection at the time of the visit. The “ringing in ears*” and “other sensations**” ratings were from the same individual within one sp/ppTMS session. TBS = Theta Burst Stimulation, Sp/ppTMS = single and paired pulse transcranial magnetic stimulation.

### Severity and specific symptoms

In TBS sessions, no “marked” or “severe” symptoms were reported. Of thirteen post-TBS AEs, twelve (92.3%) were rated “minimal’ or “mild” with one described as “moderate”. There were twenty-two post-sp/ppTMS AEs: twenty (90.9%) were rated “minimal” or “mild” and two were “moderate” or “marked”. Proportions of minimal/mild AEs did not differ between TBS and sp/ppTMS (*p* = 1.0). Specific symptoms were comparable (Table 2).

### Adverse event rates: healthy vs. affected children

Healthy control participants reported AEs in 11.5% of TBS vs. 5.3% of sp/ppTMS sessions (*p* = 0.67) (Table 3). Children with neurological diagnoses reported AEs in 8.3% of TBS vs. 14.3% of sp/ppTMS sessions (*p* = 0.72).

**Table 3:** **Healthy control participant characteristics and adverse events**.

Participant characteristics	TBS protocols (*n* = 52)	Sp/ppTMS protocols (*n* = 19)	*p*-value
Age, mean (SD)	12.6 (2.8)	12.1 (3.2)	0.44
Sex (% male)	51.9%	47.4%	0.79
% Sessions with adverse events (95% CI)	11.5% (4.4–23.4%)	5.3% (0.13–26.0%)	0.67

*CI = confidence interval, SD = standard deviation, TBS = Theta Burst Stimulation, Sp/ppTMS = single and paired pulse transcranial magnetic stimulation*.

### Adverse event rates: sham vs. active TBS

In a small sample of participants who received either active or sham cTBS (Wu et al., 2014), no difference was detected between sham vs. active TBS (*p* = 1.0).

### Predictors of AEs

No predictors of the odds of an AE were identified after data relating to all participants (Age, Sex, Diagnosis, RMT, mode of TMS) were entered into a backward logistic regression analysis.

## Discussion

In this brief report, we found no greater rate of adverse events of TBS compared to sp/ppTMS in a large cohort of pediatric subjects. The majority of AEs reported were classified minimal or mild with no severe or serious AE such as seizure. Headache was the most commonly reported specific AE in both groups. These findings represent the largest published sample analyzed to address an important gap in the safety literature regarding youth who have underwent TBS and contribute to other safety studies of TBS (Oberman et al., 2011; Wu et al., 2012; Maizey et al., 2013). The findings of this study are reassuring with regards of continued judicious use of similar TBS methods in youth.

The purpose of comparing TBS-induced AE rates to that of sp/ppTMS is because single and paired-pulse stimulations have been suggested to be “minimal risk” in children (Gilbert et al., 2004). The most recent international TMS Consensus Group “cautiously conclude that single-pulse and paired-pulse TMS in pediatrics is safe for children two years and older (Rossi et al., 2009)”. However, not all local IRB or ethics boards may agree with this statement. In the Code of Federal Regulations Section 46.102 of the United States of America, Minimal Risk means that the “probability and magnitude of harm or discomfort anticipated in the research are not greater in and of themselves than those ordinarily encountered in daily life or during the performance of routine physical or psychological examination or tests”. According to this definition, sp/ppTMS may be considered as Minimal Risk for the following reasons. First, children have rated the sp/ppTMS experience more enjoyable than several common life events (long car ride, throwing up, go to dentist, shot at the doctor’s) (Garvey et al., 2001). Second, like other medical tests commonly used in pediatric patients (e.g., brain MRI, computer tomography, nerve conduction study and EMG), TMS delivers “energy” into the human body. Contrarily, one can certainly argue that healthy children would not otherwise receive any of these medical tests and therefore TMS participation might constitute greater than minimal risk. Ultimately when working with the IRB/ethics board in developing any pediatric TMS (sp/pp or rTMS) study, it is critical to concisely present the TMS technology, scientific background/rationale, screening procedure (Rossi et al., 2011) and safety monitoring (Rossi et al., 2009; Krishnan et al., 2015) for the proposed study. As more pediatric TMS safety data emerges, it may become easier for investigators and IRB/ethical boards to objectively decide whether the proposed study is safe to proceed.

The two primary limitations of the study are sample size, and the timing of AE assessments after both sp/ppTMS and TBS in TBS protocols. Verifying small differences in rates of AEs may not be feasible without much larger samples. Using our data, we estimate approximately 4,400 children would be needed per a group to have an 80% power to detect this ~2% difference using an alpha of 0.05. AE assessments at the end of the session captured added spTMS plus TBS effects as participants also received spTMS for TBS sessions. Our finding of comparable AE rates thus suggests that the majority of AEs in TBS sessions occurred may be due to spTMS. This is further supported by findings in the sham-controlled study (Wu et al., 2014) which, consistent with the adult data (Maizey et al., 2013), were equivalent in the true and sham TBS arms. While the AE rates may also have been influenced by differences in age and case mix, the negative regression analysis suggests these effects were, at most, small. Another significant limitation is that we pooled the safety data from several studies over a five year period. Therefore, this analysis represents a heterogeneous group, including typically developing youth as well as affected pediatric population across different TMS protocols. Only one of our studies in this time period employed a sham TBS stimulation, therefore, we analyzed the data separately. Although we did not detect a difference in the sham vs. active TBS AE rates, this analysis is limited by the small sample size (*n* = 10) (Wu et al., 2014). Due to the same issue of small sample size, another concern that cannot be fully addressed in this report is the effect(s) of concurrent neuropsychiatric medication(s) on TBS safety. At the time of TBS sessions, fifteen participants were actively taking neuropsychiatric medications, some of which are known to lower seizure threshold (Pisani et al., 2002) and change cortical excitability (Ziemann et al., 2014). Given that some children were on medications that can potentially lower seizure threshold, it is encouraging that no one developed any TBS-induced seizures. Finally, the epilepsy exclusionary criterion is a limiting factor. Although available data suggests that seizure induction during rTMS for epilepsy patients is relatively low, most of the data are from adult populations (Bae et al., 2007). As TMS is increasingly used in epilepsy research, the risk of seizure provocation by various forms of rTMS, including TBS, in children with epilepsy is a knowledge gap that needs to be addressed.

TBS and other forms of rTMS hold promise for future pediatric neurophysiologic studies (Oberman et al., 2010, 2014; Damji et al., 2013) and clinical trials (Kirton et al., 2008; Wu et al., 2014). With comprehensive ongoing safety monitoring, published frequencies, and subthreshold intensities, further investigation of TBS within previously reported parameters in children appears to confer no greater risk than single and paired-pulse TMS.

## Conflict of interest statement

The authors declare that the research was conducted in the absence of any commercial or financial relationships that could be construed as a potential conflict of interest.
